# Supplementary figures and images for: VEGF Induces Expression of Genes That Either Promote or Limit Relaxation of the Retinal Endothelial Barrier
Source: Int J Mol Sci. 2023 Mar 29;24(7):6402. doi: 10.3390/ijms24076402 (PMC10094353; doi:10.3390/ijms24076402)

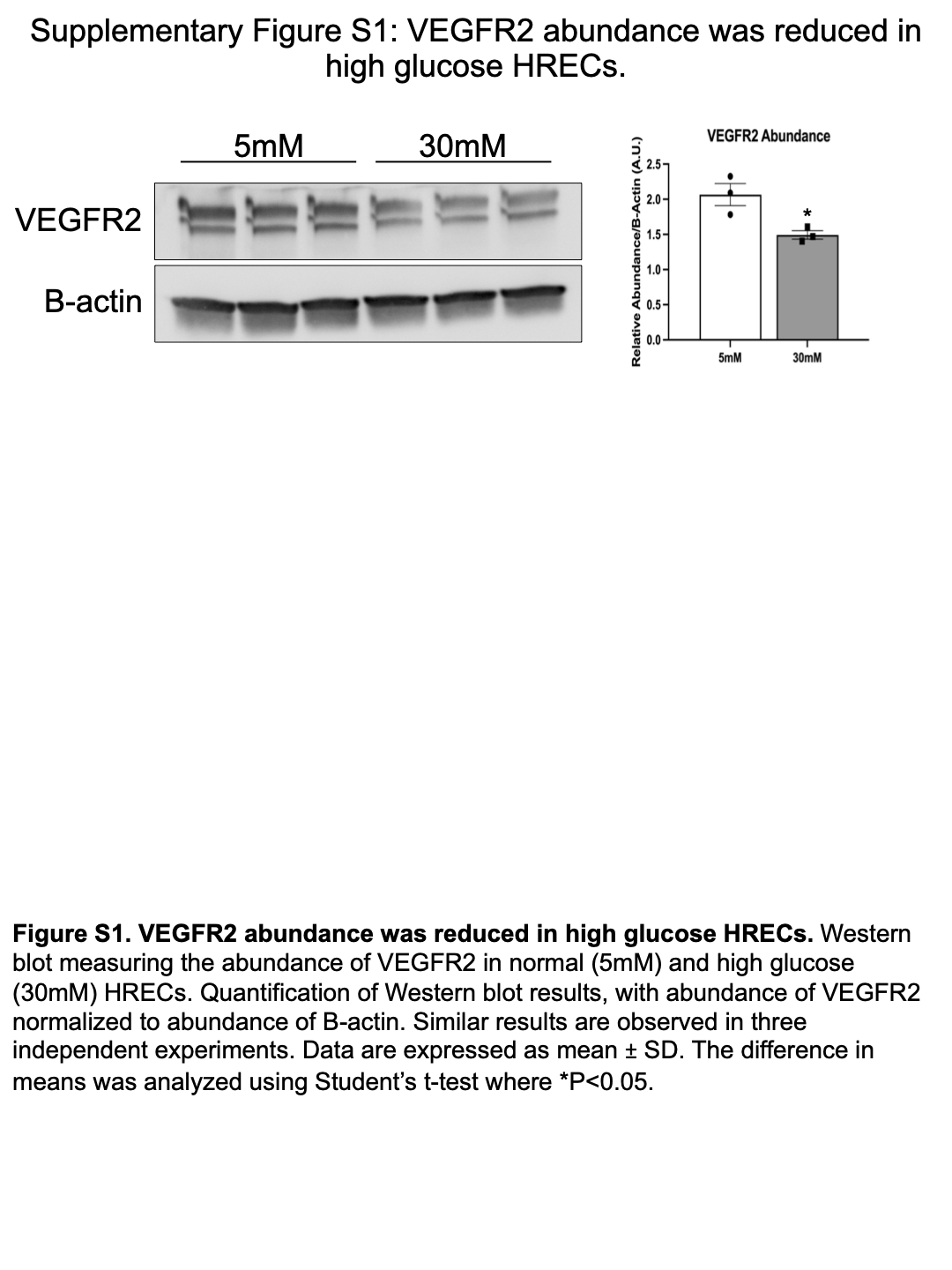

Supplement: Supplementary file 1 [file ijms-24-06402-s001.zip › Supplementary Figure S1.tif]

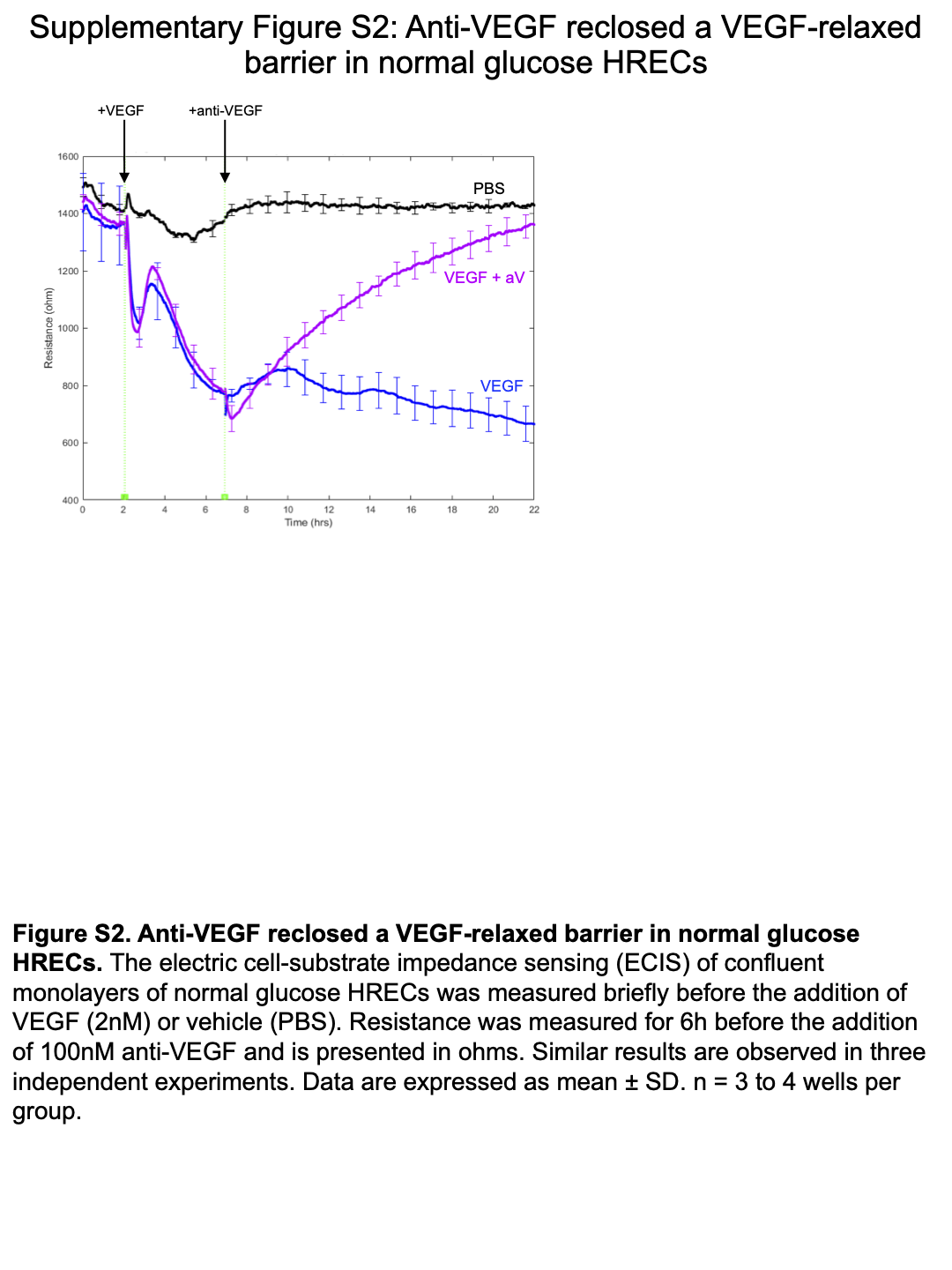

Supplement: Supplementary file 1 [file ijms-24-06402-s001.zip › Supplementary Figure S2.tif]

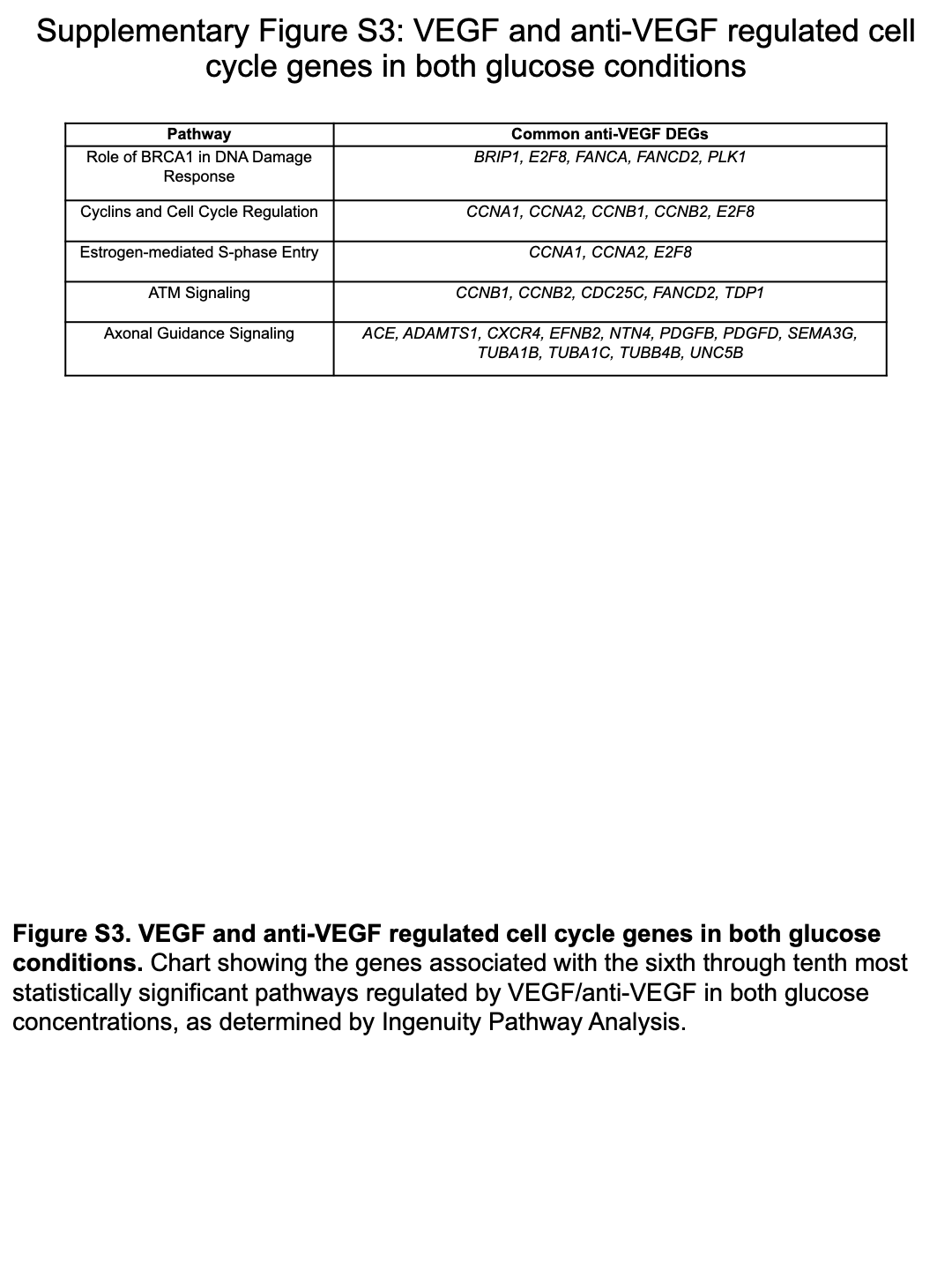

Supplement: Supplementary file 1 [file ijms-24-06402-s001.zip › Supplementary Figure S3.tif]

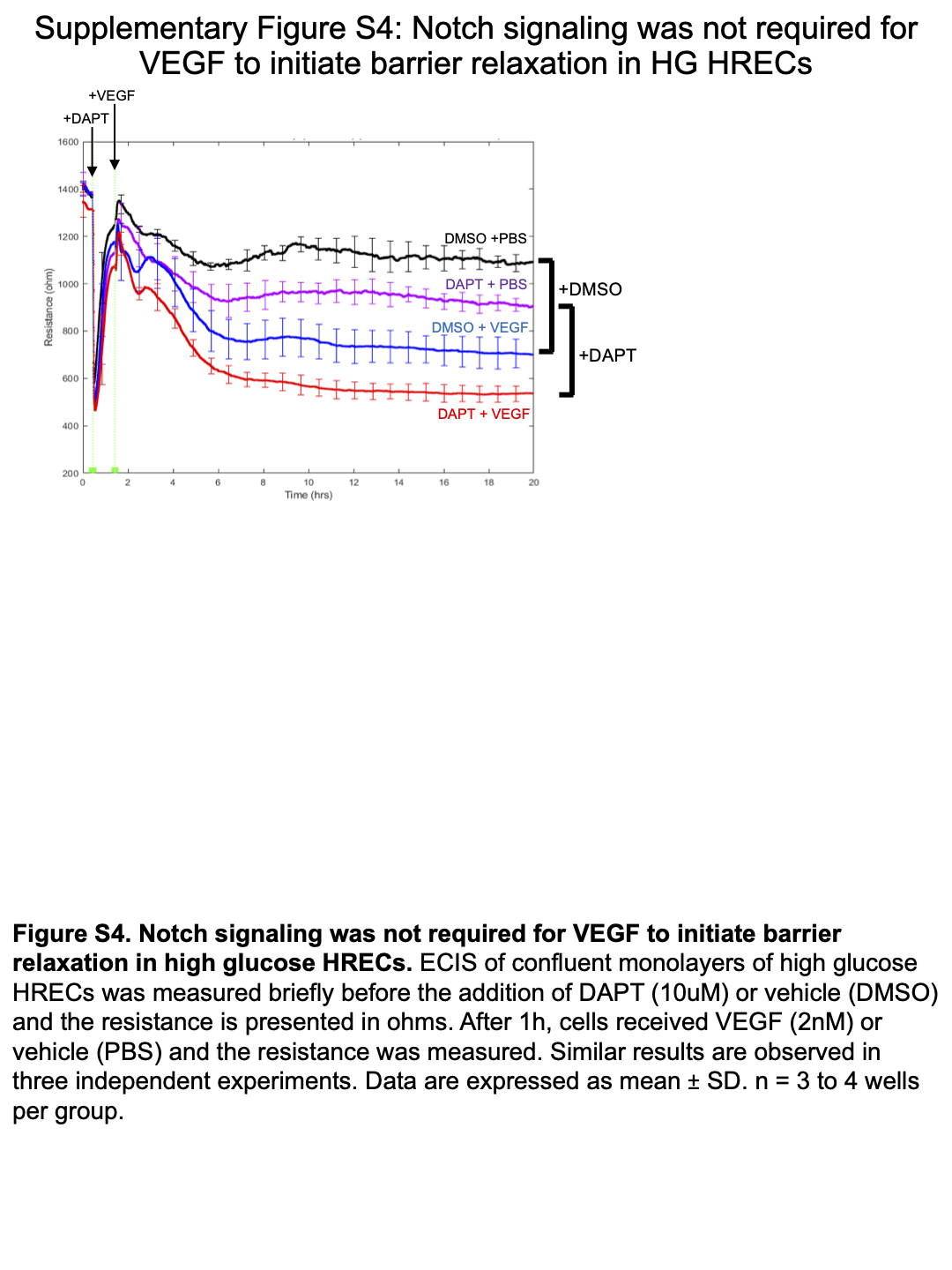

Supplement: Supplementary file 1 [file ijms-24-06402-s001.zip › Supplementary Figure S4.tif]

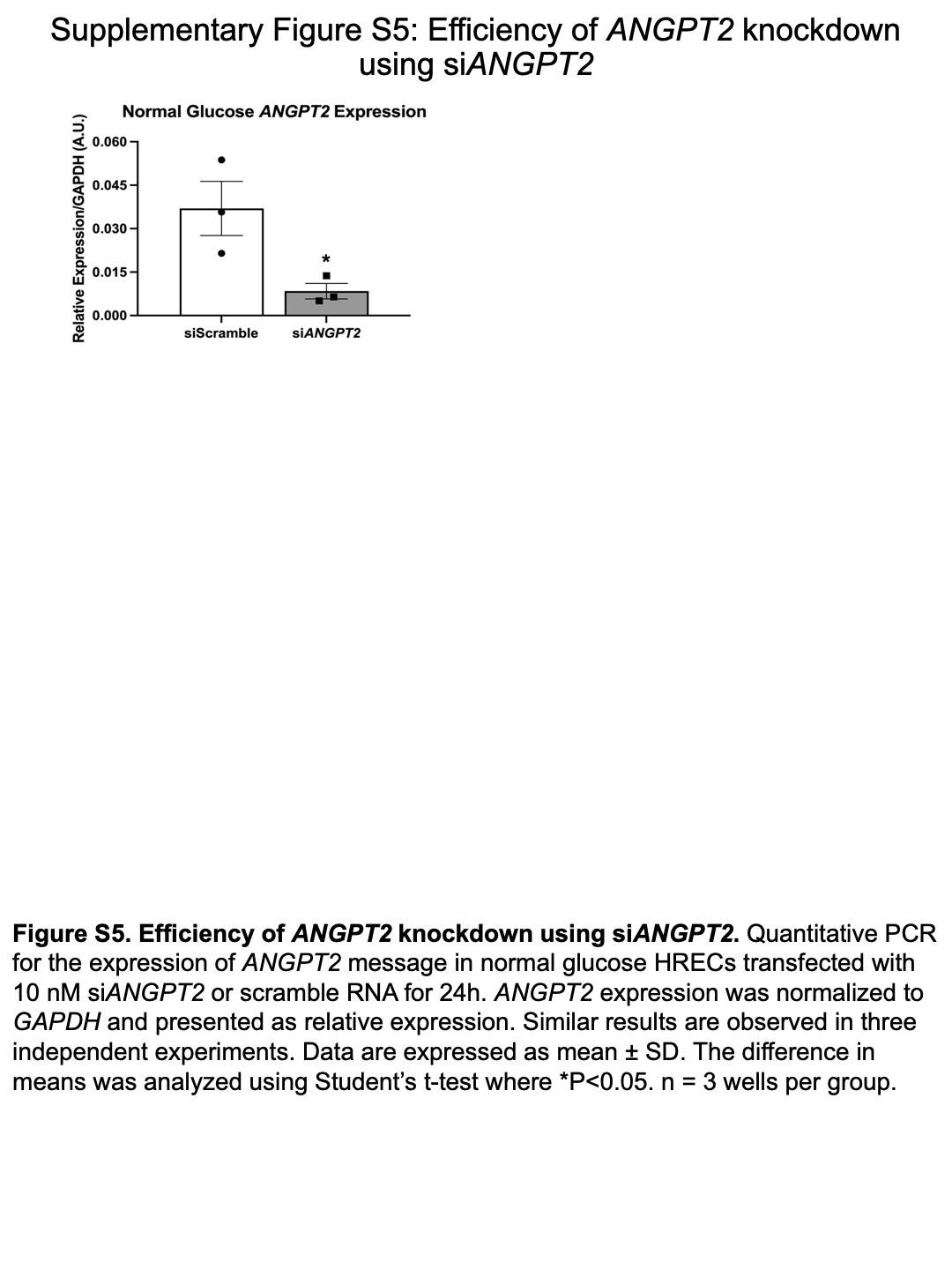

Supplement: Supplementary file 1 [file ijms-24-06402-s001.zip › Supplementary Figure S5.tif]

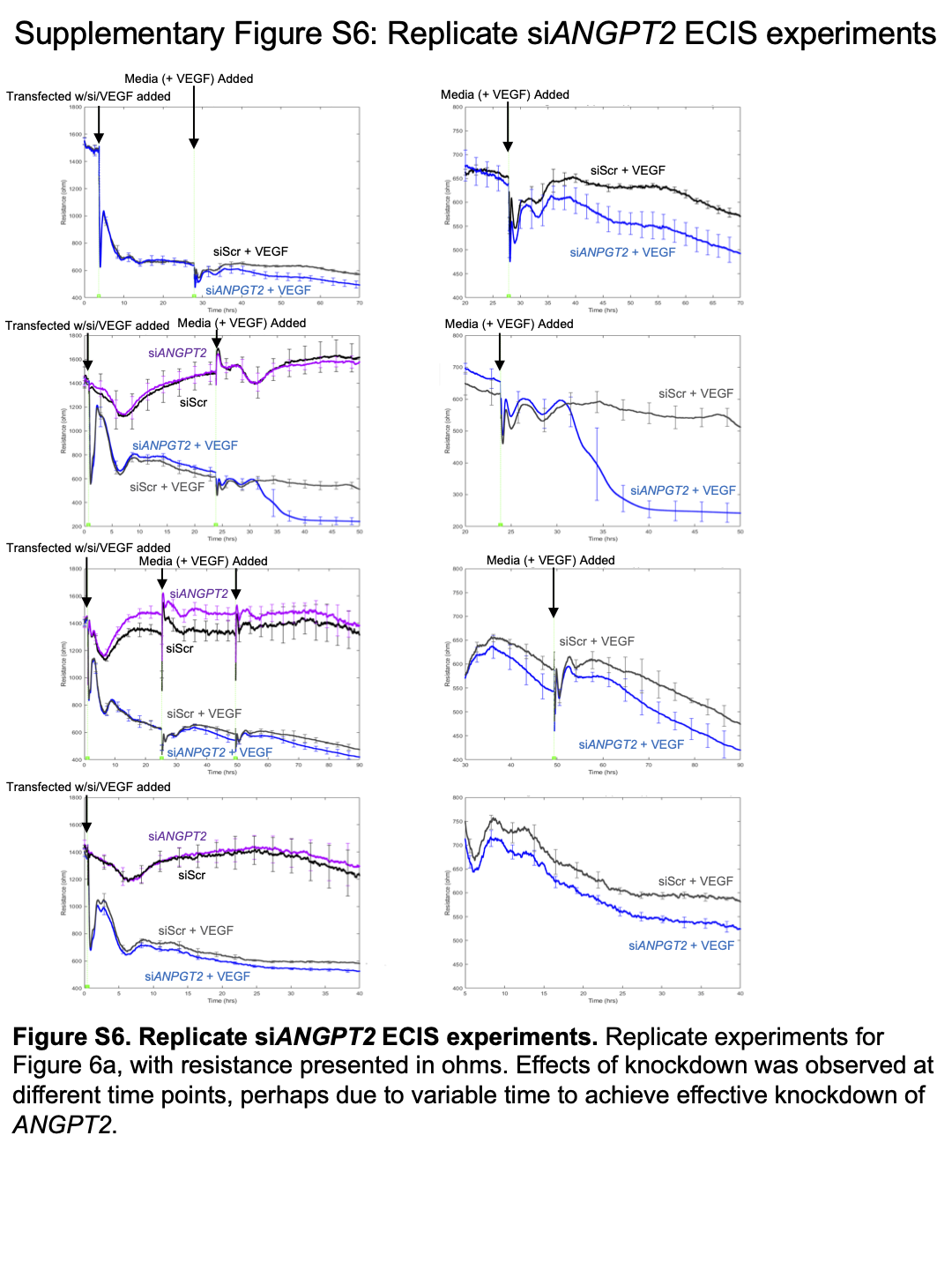

Supplement: Supplementary file 1 [file ijms-24-06402-s001.zip › Supplementary Figure S6.tif]

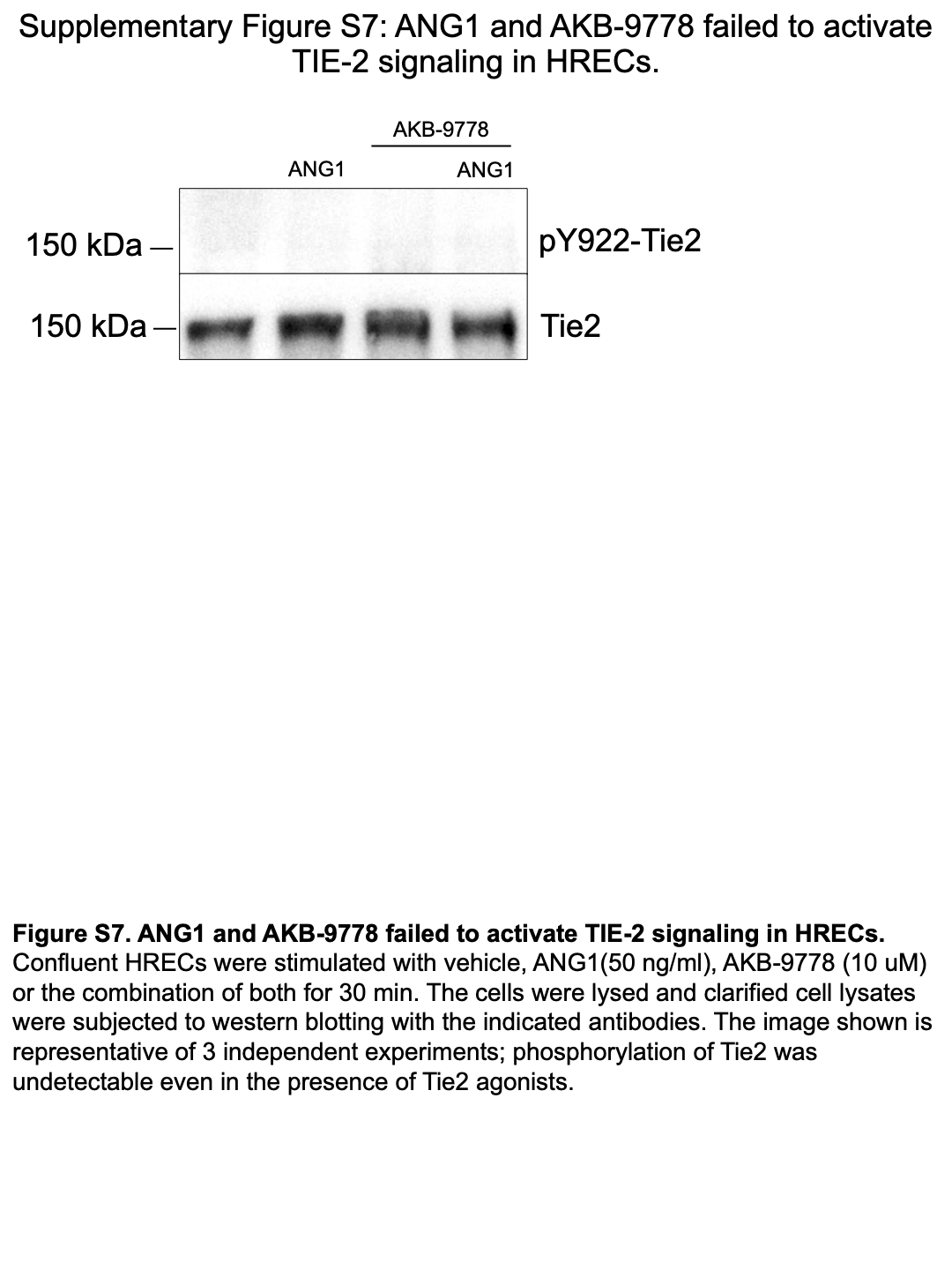

Supplement: Supplementary file 1 [file ijms-24-06402-s001.zip › Supplementary Figure S7.tif]

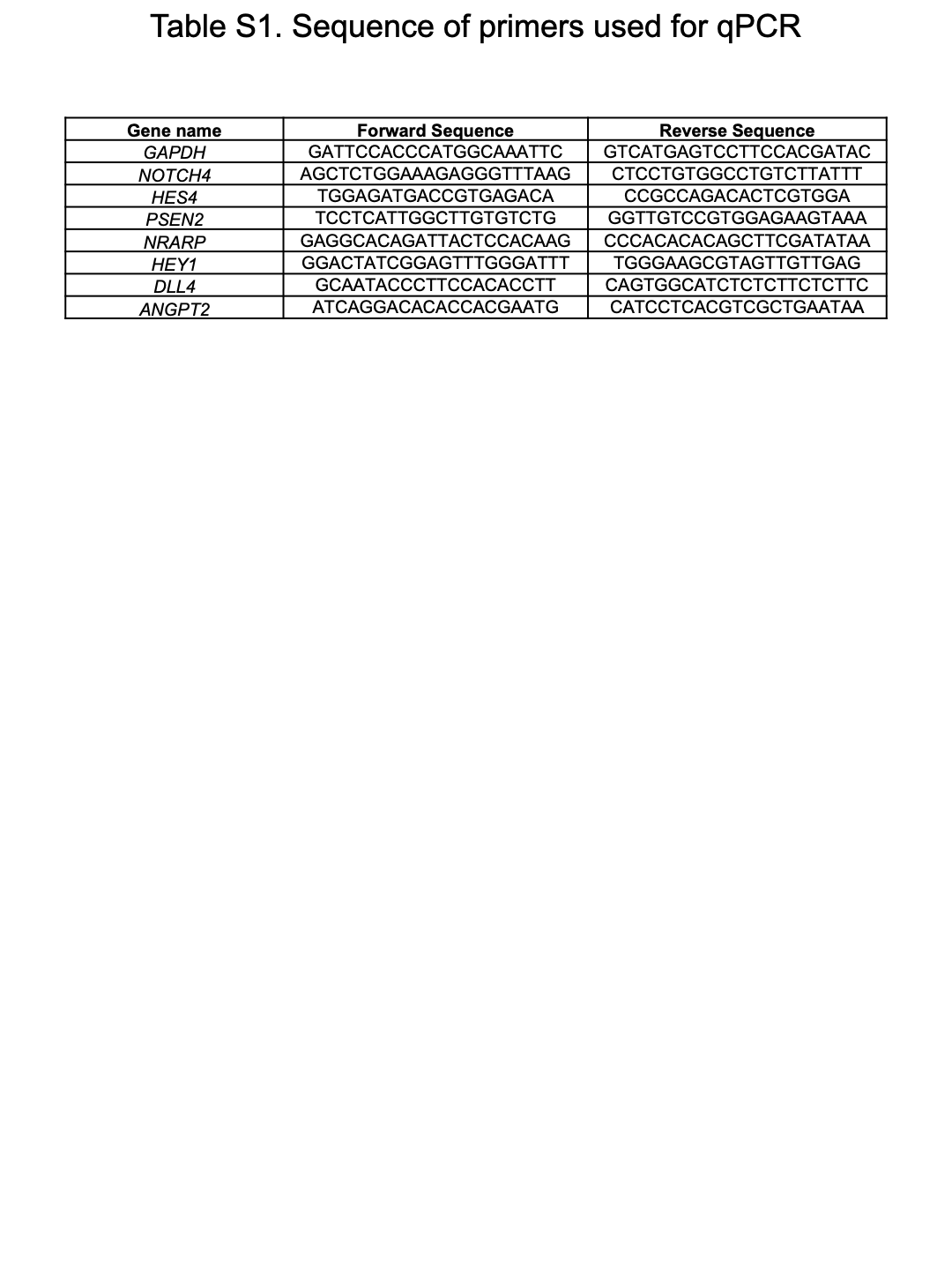

Supplement: Supplementary file 1 [file ijms-24-06402-s001.zip › Table S1.tif]
